# Responsiveness and minimal important change of Turkish pain-related psychological measures in chronic non-specific low back pain

**DOI:** 10.1080/07853890.2026.2725978

**Published:** 2026-10-05

**Authors:** Elif Esma Bayraktar, Nuray Alaca, Irmak Çavuşoğlu, Yağmur İldeniz, Asiye Koçak

**Affiliations:** a Department of Physiotherapy and Rehabilitation, Faculty of Health Sciences, Acibadem Mehmet Ali Aydinlar University, Istanbul, Türkiye; b Department of Physiotherapy and Rehabilitation, Graduate School of Health Sciences, Acibadem Mehmet Ali Aydinlar University, Istanbul, Türkiye; c Department of Physiotherapy and Rehabilitation, Faculty of Health Sciences, Kırklareli University, Kırklareli, Türkiye; d Department of Physiotherapy and Rehabilitation, Avicenna Ataşehir Hospital, Istanbul, Türkiye

**Keywords:** Fear, low back pain, pain measurement, patient-reported outcome measures, psychometrics

## Abstract

**Background:**

The longitudinal interpretability of Turkish pain-related psychological measures in chronic non-specific low back pain (NSLBP) remains uncertain. This study evaluated the responsiveness and minimal important change (MIC) of the Turkish Fear-Avoidance Beliefs Questionnaire (FABQ), Tampa Scale of Kinesiophobia (TSK) and Pain Catastrophizing Scale (PCS).

**Methods:**

In this prospective, single-arm measurement-property study, 110 adults with chronic NSLBP completed baseline and 4-week assessments during a standardized rehabilitation program. Distribution-based responsiveness was assessed using effect size and standardized response mean. Associations with Global Rating of Change (GRC)-defined improvement were examined using correlations and receiver operating characteristic analysis. MIC uncertainty was estimated by stratified bootstrap resampling with 20,000 iterations.

**Results:**

Fifty-two participants were classified as improved and 58 as not improved. FABQ change scores were strongly correlated with GRC and showed excellent discrimination (area under the curve (AUC) = 0.943–0.977). GRC-defined MIC estimates (95% bootstrap confidence interval (CI)) were 5 (5–5) for FABQ-Physical Activity and 7 (6–8) for FABQ-Work. PCS correlations and discrimination were more modest (AUC = 0.647–0.700); MIC estimates ranged from 2 to 6 points, with the widest interval for PCS-Total [6 (3–11)]. TSK change was unrelated to GRC (rho = 0.014; AUC = 0.508), and no MIC was estimated.

**Conclusions:**

In this rehabilitation context, FABQ change scores corresponded most strongly to GRC-defined improvement. PCS MIC estimates were less precise, whereas TSK change did not distinguish global improvement. These estimates are context-specific and require external validation.

## Introduction

Low back pain (LBP) remains a substantial global health burden, affecting approximately 619 million people worldwide in 2020, with the number of cases projected to reach 843 million by 2050 [1]. Most cases are classified as non-specific low back pain (NSLBP), and persistent symptoms are rarely explained by structural or biomedical factors alone [2,3]. In chronic NSLBP, pain-related beliefs and emotional and behavioural responses may influence disability, rehabilitation engagement and perceived improvement. Assessing these factors is therefore important for understanding recovery beyond changes in pain intensity or physical function.

Fear-avoidance beliefs, kinesiophobia and pain catastrophizing are commonly assessed using the Fear-Avoidance Beliefs Questionnaire (FABQ), Tampa Scale of Kinesiophobia (TSK) and Pain Catastrophizing Scale (PCS), respectively [4–7]. The FABQ assesses beliefs that physical activity or work may exacerbate pain or cause harm [7]; the TSK assesses fear of movement or reinjury [6]; and the PCS assesses exaggerated negative cognitive and emotional responses to pain [5]. Because these instruments measure distinct but related constructs, they may differ in their ability to detect clinically meaningful change during rehabilitation.

For patient-reported outcome measures used in longitudinal studies, validity and reliability alone do not establish whether an instrument can detect change over time. Responsiveness reflects the ability of an instrument to detect change in the construct it is intended to measure [8,9]. The minimal important change (MIC), in turn, represents the smallest change that patients perceive as important. Evaluating responsiveness together with anchor-based MIC estimates can therefore support the clinical interpretation of changes in FABQ, TSK and PCS scores.

Pain-related psychological constructs may be influenced by cultural beliefs, expectations about pain, attitudes towards movement and healthcare experiences. Therefore, responsiveness findings and MIC estimates obtained in one language or cultural context may not be directly applicable to another. Turkish versions of the FABQ, TSK and PCS have been translated and evaluated for validity and/or reliability in Turkish-speaking populations [10–14]. However, these studies primarily examined cross-sectional psychometric properties or test–retest reliability rather than anchor-based responsiveness and MIC estimates in patients with chronic NSLBP.

Previous studies have investigated responsiveness and reported MIC estimates for the FABQ and PCS in individuals with chronic LBP undergoing multidisciplinary rehabilitation, while a recent study evaluated the Persian versions of the FABQ, TSK and PCS in patients with chronic NSLBP [15–17]. Nevertheless, evidence for the Turkish versions remains limited, and few studies have directly compared all three instruments within the same cohort using a common patient-reported anchor. Such a comparison may clarify differences in their ability to detect clinically important change and support the selection and interpretation of these measures in Turkish-speaking patients with chronic NSLBP.

Therefore, this study aimed to evaluate and compare the responsiveness of the Turkish versions of the FABQ, TSK and PCS in patients with chronic NSLBP and to derive anchor-based MIC estimates using the Global Rating of Change (GRC) as the anchor.

## Methods

### Study design and participants

This prospective, single-arm measurement-property study was conducted within a rehabilitation program intended to induce clinical change. Participants were adults referred to the physical therapy and rehabilitation outpatient clinic of Avicenna Ataşehir Hospital, Istanbul, Turkey, for conservative management of chronic NSLBP. Questionnaire data were collected between December 2024 and March 2026. The study was prospectively registered (NCT06271447).

The study protocol was approved by the Acibadem Mehmet Ali Aydinlar University Medical Research Ethics Committee (ATADEK; approval number: 2023-21/705; approval date: 11 January 2024). All participants provided written informed consent before enrolment, and the study was conducted in accordance with the principles of the Declaration of Helsinki.

Adults aged 18–65 years with chronic NSLBP were eligible for inclusion. Chronic NSLBP was defined as LBP persisting for more than 12 weeks without a recognizable specific pathoanatomical cause, such as fracture, tumour, infection, inflammatory disease, radiculopathy or previous lumbar surgery. Participants were required to be able to read and understand Turkish and to have sufficient cognitive ability to complete self-reported questionnaires. Cognitive status was screened using the Standardized Mini-Mental Test, and a score of ≥24 was required for inclusion.

Exclusion criteria were radiculopathy; spinal infection, tumour, osteoporosis, vertebral fracture, recent trauma or lumbar surgery; systemic disease affecting participation or interpretation; cognitive impairment; myocardial infarction or cerebrovascular event within 12 weeks; previous structured exercise therapy or pain neuroscience education for LBP; refusal of treatment; or inability to comply with the program. Patients who had received an intramuscular analgesic injection for severe LBP within the preceding month were also excluded because they followed a separate clinical care pathway rather than the standardized rehabilitation program.

A total of 110 patients with chronic NSLBP were included in the final analysis. Assessments were performed at baseline (T0), before the rehabilitation program, and at follow-up (T1), after completion of the 4-week rehabilitation program (Figure 1).

**Figure 1.**
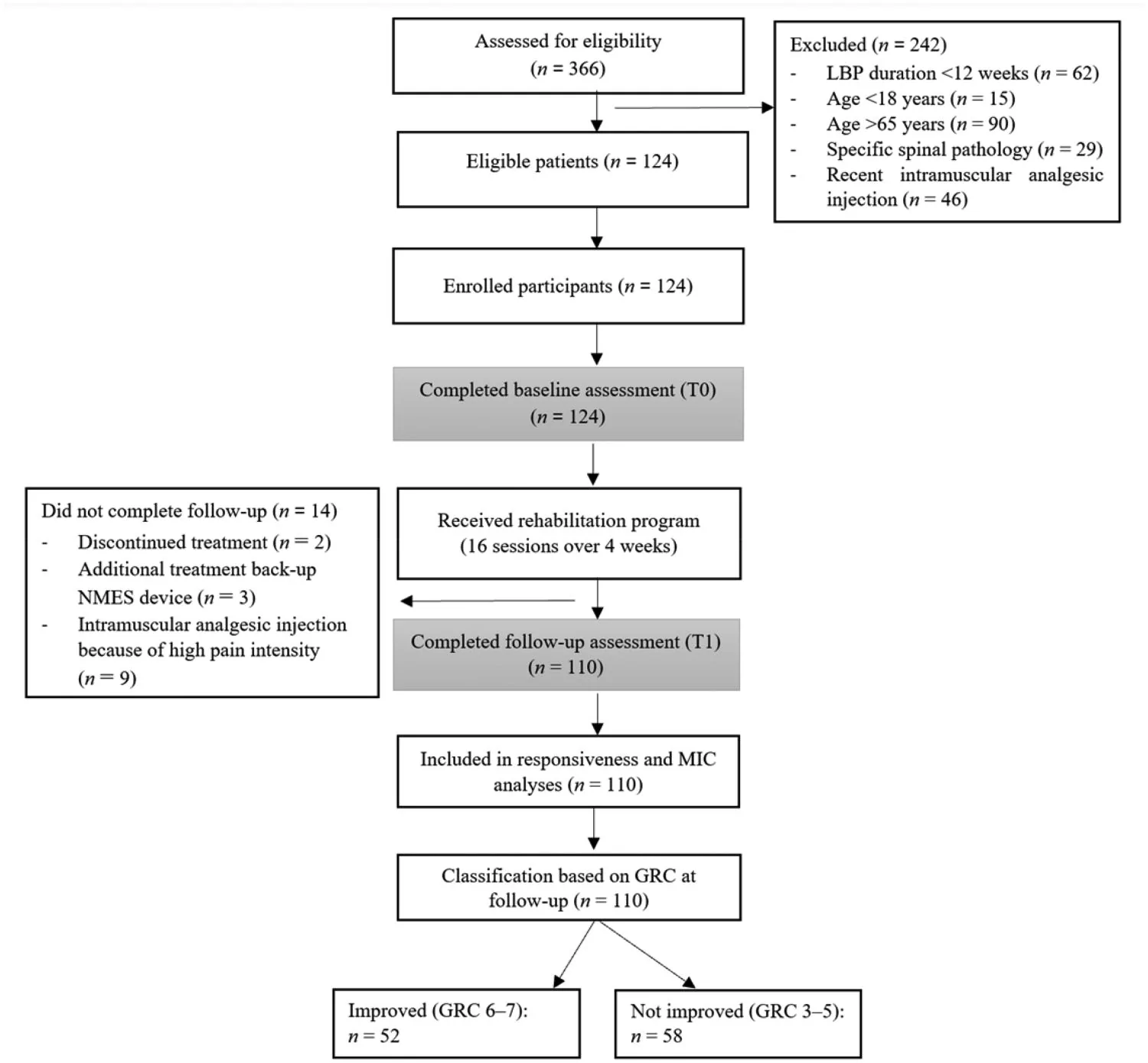
Flow diagram of participant recruitment, reasons for non-completion of follow-up, and classification according to the Global Rating of Change (GRC).

### Intervention

Participants completed a conventional physiotherapy program consisting of 16 supervised sessions over four weeks, with four 60-minute sessions delivered each week. The program included lumbar stabilization exercises, trunk strengthening, lower-limb flexibility training and postural education. As part of the standardized rehabilitation program, all 110 participants included in the analysis received physician-prescribed electrotherapy during each of the 16 treatment sessions. The electrotherapy protocol comprised superficial heat using a hot pack for approximately 20 min, conventional transcutaneous electrical nerve stimulation (TENS) at 80–100 Hz with a pulse duration of approximately 100 μs for 20 min, and therapeutic ultrasound applied to the lumbar paraspinal region at 1 MHz and 1–1.5 W/cm^2^ for approximately 5 min. Similar multimodal physiotherapy approaches have been used in chronic LBP populations in previous clinical studies [18,19]. Exercise-based rehabilitation and education were considered the core components of the program, while electrotherapy was delivered as a standardized adjunctive component of routine care, consistent with contemporary recommendations emphasizing active management for chronic LBP [20].

The rehabilitation protocol also included a structured pain-related cognitive education component delivered once weekly over the 4-week program, with each session lasting approximately 45 min. The education addressed pain neurophysiology, differences between acute and chronic pain, central and cortical mechanisms involved in persistent pain, pain-related fear and avoidance beliefs, ergonomic principles, and safe movement strategies. The aim was to help patients reconceptualize pain within a biopsychosocial framework, reduce the perceived threat value of pain, and support adaptive beliefs about movement. This content was consistent with pain neuroscience education principles, which emphasize that pain is a modifiable protective output rather than a direct marker of tissue damage [21–23].

The program was delivered using the same core protocol by two licensed physiotherapists, each with at least five years of clinical experience. Neither physiotherapist participated in the outcome assessments. Electrotherapy was considered part of routine clinical care rather than a study-controlled intervention. The program was used to generate change for measurement-property evaluation, not to test treatment effectiveness [15,17].

### Outcome measures

Sociodemographic and clinical characteristics, including age, sex, body mass index, marital status, education level, smoking status, occupation and pain duration, were recorded at baseline.

Pain intensity was assessed using a 10-cm Visual Analogue Scale (VAS) separately at rest and during activity. The scale was anchored by ‘no pain’ and ‘worst imaginable pain,’ and participants marked their perceived pain intensity on the line. The distance from the left anchor was measured in centimetres, with higher scores indicating greater pain intensity [24,25]. Pain quality was assessed using the Short-Form McGill Pain Questionnaire (SF-MPQ), which provides sensory, affective and total pain descriptor scores, with higher scores indicating greater pain severity [26,27]. Disability was assessed using the Oswestry Disability Index [28,29], expressed as a percentage, with higher scores indicating greater disability [7].

The 16-item FABQ assesses fear-avoidance beliefs related to Physical Activity (items 2–5; range, 0–24) and Work (items 6, 7, 9–12 and 15; range, 0–42) [7,10,11]. Analyses were restricted to these validated subscales. All participants completed the standard Turkish FABQ without a not-applicable option. Non-working participants received no standardized frame of reference for the work-related items, and the basis on which they interpreted those items was not recorded.

The TSK was used to assess fear of movement or reinjury. The scale consists of 17 items scored on a four-point Likert scale, ranging from ‘strongly disagree’ to ‘strongly agree.’ After reverse scoring of the relevant items, total scores range from 17 to 68, with higher scores indicating greater kinesiophobia. The Turkish version of the TSK has previously demonstrated test–retest reliability [6,14].

The PCS was used to assess catastrophic thinking related to pain. The scale consists of 13 items scored on a 0–4 Likert scale and provides Rumination, Magnification, Helplessness and Total scores. Higher scores indicate greater pain catastrophizing. The Turkish version of the PCS has previously been validated in Turkish-speaking populations [5,12,13].

At follow-up, participants completed the Turkish GRC. The exact question was ‘Tedavinin başlangıcıyla karşılaştırıldığında şu anki durumunuzu nasıl tanımlarsınız?’ (‘How would you describe your current condition compared with the beginning of treatment?’). Response options were 7, ‘Çok daha iyi’ (‘much better’); 6, ‘Daha iyi’ (‘better’); 5, ‘Biraz daha iyi’ (‘a little better’); 4, ‘Değişiklik yok’ (‘no change’); 3, ‘Biraz kötü’ (‘a little worse’); 2, ‘Daha kötü’ (‘worse’); and 1, ‘Çok daha kötü’ (‘much worse’). Scores of 6–7 defined improvement, whereas scores of 3–5 defined not-improved status. Because the GRC assessed global condition rather than a specific psychological construct, MIC estimates were interpreted as GRC-defined and context-specific [30].

### Statistical analysis

Statistical analyses were performed using IBM SPSS Statistics for Windows, version 21.0 (IBM Corp., Armonk, NY), and Jamovi, version 1.2.27 (The jamovi project, Sydney, Australia). Statistical significance was set at *p* ≤ 0.05, two-tailed.

Continuous variables were summarized as mean ± standard deviation (SD), and categorical variables were summarized as frequency and percentage. Normality was evaluated using the Shapiro–Wilk test together with skewness and kurtosis values. Because formal normality tests may be sensitive in moderate sample sizes, skewness values within ±2 and kurtosis values within ±7 were considered to indicate approximate normal distribution [31].

Baseline groups were compared using independent-samples *t* tests for continuous variables and chi-square or Monte Carlo exact tests for categorical variables, as appropriate. The Monte Carlo exact test used 500,000 samples. Within-group change was examined using paired-samples *t*-tests. Change scores were calculated as T0–T1, so positive values indicated improvement. Floor or ceiling effects were defined as more than 15% of participants scoring at the respective scale limit [9,32].

Distribution-based responsiveness was quantified using effect size (mean change/baseline SD) and standardized response mean (mean change/SD of change). Anchor correspondence was evaluated using Spearman’s correlations between change scores and GRC. Receiver operating characteristic (ROC) analysis estimated discrimination between GRC-defined groups; MIC was the threshold maximizing the Youden Index. Stratified bootstrap resampling with 20,000 iterations preserved group sizes, and the 2.5th and 97.5th percentiles of the optimal-threshold distribution defined the 95% confidence interval (CI). MIC was not estimated when anchor correlation or discrimination was negligible. FABQ-Work analyses were repeated in currently employed participants [32,33].

### Sample size considerations

Sample size was determined with reference to previous responsiveness and MIC studies evaluating pain-related psychological measures in spinal pain populations and to COSMIN (COnsensus-based Standards for the selection of health Measurement INstruments) recommendations for studies assessing measurement properties of patient-reported outcome measures. Previous clinimetric studies evaluating the FABQ, TSK and PCS or related pain-related outcome measures have commonly included samples of approximately 50–100 participants, and COSMIN guidance considers sample sizes of at least 100 participants very good for evaluating measurement properties. Therefore, the total sample of 110 was considered adequate with reference to previous responsiveness studies and COSMIN guidance. Precision of ROC-derived MIC estimates was evaluated using bootstrap CIs, and external validation was considered necessary [9,15,17].

## Results

### Participant flow and baseline characteristics

Of 366 patients screened, 242 were excluded and 124 enrolled. Fourteen participants (11.3%) did not complete follow-up because they discontinued treatment or received additional treatment during the study (Figure 1). The remaining 110 completed all 16 sessions and provided complete baseline and follow-up questionnaire data; no item-level imputation was performed. Fifty-two were classified as improved (GRC 6–7) and 58 as not improved (GRC 3–5).

Baseline demographic and clinical characteristics were comparable between the improved and not improved groups (Table 1). No statistically significant between-group differences were observed for age, sex, body mass index, marital status, education level, smoking status, occupation, pain duration, pain intensity, disability or baseline FABQ, TSK and PCS scores.

**Table 1.** Baseline demographic and clinical characteristics of participants according to GRC-based improvement status.

Variable	Total (*n* = 110)	Not improved (*n* = 58)	Improved (*n* = 52)	*p* Value
Age (years)	36.36 ± 10.01	35.88 ± 9.65	36.90 ± 10.47	0.594
Female sex, *n* (%)	44 (40.0)	23 (39.7)	21 (40.4)	0.938^a^
Body mass index (kg/m^2^)	25.82 ± 1.49	25.85 ± 1.40	25.79 ± 1.60	0.858
Married, *n* (%)	72 (65.5)	37 (63.8)	35 (67.3)	0.699^a^
Education level, *n* (%)				0.062^b^
No formal education	3 (2.7)	3 (5.2)	0 (0.0)	
High school	26 (23.6)	11 (19.0)	15 (28.8)	
University	74 (67.3)	38 (65.5)	36 (69.2)	
Postgraduate	7 (6.4)	6 (10.3)	1 (1.9)	
Smoker, *n* (%)	21 (19.1)	11 (19.0)	10 (19.2)	0.972^a^
Occupation, *n* (%)				0.079^a^
Office worker	25 (22.7)	16 (27.6)	9 (17.3)	
Other employed	49 (44.5)	20 (34.5)	29 (55.8)	
Not working	36 (32.7)	22 (37.9)	14 (26.9)	
*Clinical characteristics and outcome measures*				
Pain duration (months)	12.40 ± 6.94	12.16 ± 6.62	12.66 ± 7.34	0.703
SF-MPQ sensory score	7.25 ± 1.42	7.36 ± 1.40	7.12 ± 1.44	0.364
SF-MPQ affective score	3.48 ± 1.10	3.59 ± 1.09	3.35 ± 1.10	0.254
SF-MPQ total score	10.72 ± 2.35	10.95 ± 2.30	10.46 ± 2.40	0.279
Pain intensity at rest, VAS (cm)	4.20 ± 0.81	4.28 ± 0.80	4.12 ± 0.83	0.285
Pain intensity during activity, VAS (cm)	5.29 ± 0.85	5.36 ± 0.83	5.22 ± 0.87	0.391
Oswestry Disability Index (%)	26.80 ± 5.76	26.93 ± 5.40	26.65 ± 6.19	0.802
Tampa Scale of Kinesiophobia	40.06 ± 7.23	41.12 ± 6.91	38.88 ± 7.47	0.106
FABQ-Physical Activity	15.21 ± 2.03	15.28 ± 1.88	15.13 ± 2.21	0.718
FABQ-Work	22.60 ± 2.91	22.64 ± 2.84	22.56 ± 3.02	0.886
PCS-Rumination	6.27 ± 2.22	6.45 ± 2.18	6.06 ± 2.26	0.358
PCS-Magnification	4.06 ± 1.37	4.12 ± 1.33	4.00 ± 1.43	0.647
PCS-Helplessness	10.86 ± 3.03	11.02 ± 2.83	10.69 ± 3.26	0.577
PCS-Total	21.19 ± 6.42	21.59 ± 6.13	20.75 ± 6.77	0.498

FABQ: Fear-Avoidance Beliefs Questionnaire; GRC: Global Rating of Change; PCS: Pain Catastrophizing Scale; SF-MPQ: Short-Form McGill Pain Questionnaire; VAS: Visual Analogue Scale.

Values are presented as mean ± standard deviation for continuous variables and *n* (%) for categorical variables. Between-group comparisons were performed using independent-samples *t*-tests for continuous variables and chi-square tests for categorical variables, except for education level, which was evaluated using a Monte Carlo exact test with 500,000 samples.

^a^ Chi-square test.

^b^ Monte Carlo exact test.

### Within-group changes and distribution-based responsiveness

All clinical and psychological outcomes improved from baseline in both GRC groups (all *p* < 0.001; Table 2). FABQ and PCS changes were numerically larger in the improved group, whereas TSK change was similar between groups.

**Table 2.** Within-group changes and distribution-based responsiveness according to GRC-based improvement status.

Outcome measure	Group	Baseline	Follow-up	Mean change (95% CI)	Effect size	SRM	*p* Value
Pain intensity at rest, VAS (cm)	Not improved	4.28 ± 0.80	2.62 ± 1.39	1.66 (1.35–1.98)	2.08	1.38	<0.001
	Improved	4.12 ± 0.83	1.52 ± 1.18	2.59 (2.27–2.91)	3.13	2.26	<0.001
Pain intensity during activity, VAS (cm)	Not improved	5.36 ± 0.83	3.62 ± 1.38	1.74 (1.43–2.05)	2.09	1.46	<0.001
	Improved	5.22 ± 0.87	2.60 ± 1.11	2.62 (2.37–2.88)	3.03	2.89	<0.001
SF-MPQ affective score	Not improved	3.59 ± 1.09	2.07 ± 1.37	1.52 (1.14–1.89)	1.39	1.06	<0.001
	Improved	3.35 ± 1.10	1.25 ± 1.05	2.10 (1.76–2.43)	1.90	1.73	<0.001
SF-MPQ sensory score	Not improved	7.36 ± 1.40	5.26 ± 1.61	2.10 (1.74–2.47)	1.50	1.50	<0.001
	Improved	7.12 ± 1.44	3.96 ± 1.36	3.15 (2.74–3.57)	2.19	2.12	<0.001
SF-MPQ total score	Not improved	10.95 ± 2.30	7.33 ± 2.74	3.62 (2.97–4.27)	1.58	1.46	<0.001
	Improved	10.46 ± 2.40	5.21 ± 2.21	5.25 (4.59–5.91)	2.19	2.20	<0.001
ODI, %	Not improved	26.93 ± 5.40	22.53 ± 5.45	4.40 (3.09–5.71)	0.81	0.88	<0.001
	Improved	26.65 ± 6.19	16.37 ± 6.31	10.29 (9.14–11.43)	1.66	2.50	<0.001
TSK	Not improved	41.12 ± 6.91	37.24 ± 6.42	3.88 (3.27–4.49)	0.56	1.66	<0.001
	Improved	38.88 ± 7.47	34.96 ± 6.47	3.92 (3.29–4.55)	0.53	1.74	<0.001
FABQ-Physical Activity	Not improved	15.28 ± 1.88	13.14 ± 2.87	2.14 (1.62–2.65)	1.14	1.09	<0.001
	Improved	15.13 ± 2.21	9.23 ± 3.15	5.90 (5.50–6.31)	2.67	4.05	<0.001
FABQ-Work	Not improved	22.64 ± 2.84	19.45 ± 4.04	3.19 (2.43–3.95)	1.12	1.11	<0.001
	Improved	22.56 ± 3.02	12.77 ± 4.10	9.79 (9.19–10.39)	3.24	4.54	<0.001
PCS-Rumination	Not improved	6.45 ± 2.18	5.03 ± 2.15	1.41 (0.95–1.87)	0.65	0.81	<0.001
	Improved	6.06 ± 2.26	3.67 ± 1.87	2.38 (1.87–2.90)	1.05	1.28	<0.001
PCS-Magnification	Not improved	4.12 ± 1.33	3.03 ± 1.32	1.09 (0.78–1.39)	0.82	0.93	<0.001
	Improved	4.00 ± 1.43	2.27 ± 1.21	1.73 (1.39–2.07)	1.21	1.43	<0.001
PCS-Helplessness	Not improved	11.02 ± 2.83	7.66 ± 3.01	3.36 (2.59–4.14)	1.19	1.14	<0.001
	Improved	10.69 ± 3.26	5.48 ± 2.68	5.21 (4.52–5.91)	1.60	2.08	<0.001
PCS-Total	Not improved	21.59 ± 6.13	15.72 ± 6.34	5.86 (4.38–7.34)	0.96	1.04	<0.001
	Improved	20.75 ± 6.77	11.42 ± 5.64	9.33 (7.84–10.81)	1.38	1.75	<0.001

CI: confidence interval; FABQ: Fear-Avoidance Beliefs Questionnaire; GRC: Global Rating of Change; ODI: Oswestry Disability Index; PCS: Pain Catastrophizing Scale; SF-MPQ: Short-Form McGill Pain Questionnaire; SRM: standardized response mean; TSK: Tampa Scale of Kinesiophobia; VAS: Visual Analogue Scale.

Values are presented as mean ± standard deviation unless otherwise indicated. Mean change was calculated as baseline minus follow-up value (T0–T1), with positive values indicating improvement. Effect size was calculated as the mean change divided by the baseline standard deviation. SRM was calculated as the mean change divided by the standard deviation of the change score.

Both FABQ subscales showed large distribution-based responsiveness indices, with the largest values observed for FABQ-Work. PCS distribution-based responsiveness indices ranged from moderate to large, while TSK demonstrated substantial change despite little between-group separation.

### Anchor-based correspondence and GRC-defined MIC

FABQ change scores were strongly correlated with GRC (rho = 0.884–0.937), PCS correlations were moderate (rho = 0.371–0.467) and TSK correlation was negligible (rho = 0.014; Table 3). FABQ area under the curves (AUCs) ranged from 0.943 to 0.977, compared with 0.647–0.700 for PCS and 0.508 for TSK.

**Table 3.** Anchor-based correspondence and GRC-defined minimal important change of pain-related psychological measures.

Instrument	Anchor correlation (Spearman’s rho)	AUC (95% CI)	MIC (95% bootstrap CI)	Sensitivity	Specificity	Youden Index
TSK	0.014 (*p* = 0.881)	0.508 (0.400–0.617)	–	–	–	–
FABQ						
Physical Activity	0.884 (*p* < 0.001)	0.943 (0.901–0.984)	5 (5–5)	0.865	0.931	0.796
Work	0.937 (*p* < 0.001)	0.977 (0.953–1.000)	7 (6–8)	0.942	0.931	0.873
PCS						
Rumination	0.372 (*p* < 0.001)	0.647 (0.544–0.749)	3 (1–3)	0.500	0.741	0.241
Magnification	0.371 (*p* < 0.001)	0.648 (0.546–0.751)	2 (1–3)	0.596	0.655	0.251
Helplessness	0.467 (*p* < 0.001)	0.700 (0.601–0.798)	5 (4–6)	0.692	0.776	0.468
Total	0.431 (*p* < 0.001)	0.683 (0.583–0.782)	6 (3–11)	0.769	0.552	0.321

AUC: area under the receiver operating characteristic curve; FABQ: Fear-Avoidance Beliefs Questionnaire; GRC: Global Rating of Change; MIC: minimal important change; PCS: Pain Catastrophizing Scale; ROC: receiver operating characteristic; TSK: Tampa Scale of Kinesiophobia.

Values are presented as AUC with 95% CI and MIC with 95% bootstrap CI. Change scores were calculated as T0–T1, with positive values indicating improvement. MIC maximized the Youden Index. Stratified bootstrap resampling with 20,000 iterations preserved GRC group sizes; percentile limits defined the 95% CI. No MIC was estimated for TSK because anchor association and discrimination were negligible.

GRC-defined MIC estimates (95% bootstrap CI) were 5 (5–5) for FABQ-Physical Activity, 7 (6–8) for FABQ-Work, 3 (1–3) for PCS-Rumination, 2 (1–3) for PCS-Magnification, 5 (4–6) for PCS-Helplessness and 6 (3–11) for PCS-Total. No MIC was estimated for TSK. In employed participants (*n* = 74), FABQ-Work findings remained consistent [rho = 0.952; AUC = 0.986 (95% CI 0.965–0.999); threshold approximately 8; sensitivity 0.895; specificity 0.972].

The observed GRC distribution was: score 1, *n* = 0; score 2, *n* = 0; score 3, *n* = 7; score 4, *n* = 16; score 5, *n* = 35; score 6, *n* = 32; and score 7, *n* = 20. The GRC 3–5 group was interpreted as not improved, not stable. Because only 16 participants selected GRC = 4, measurement error was not estimated.

### Floor and ceiling effects

No floor or ceiling effect exceeded the prespecified 15% threshold at baseline or follow-up (Table 4).

**Table 4.** Floor and ceiling effects of FABQ, TSK and PCS at baseline and follow-up.

Measure	Baseline floor, *n* (%)	Baseline ceiling, *n* (%)	Follow-up floor, *n* (%)	Follow-up ceiling, *n* (%)
TSK	0 (0.0)	0 (0.0)	0 (0.0)	0 (0.0)
FABQ-Physical Activity	0 (0.0)	0 (0.0)	0 (0.0)	0 (0.0)
FABQ-Work	0 (0.0)	0 (0.0)	0 (0.0)	0 (0.0)
PCS-Rumination	0 (0.0)	0 (0.0)	1 (0.9)	0 (0.0)
PCS-Magnification	0 (0.0)	0 (0.0)	2 (1.8)	0 (0.0)
PCS-Helplessness	0 (0.0)	0 (0.0)	1 (0.9)	0 (0.0)
PCS-Total	0 (0.0)	0 (0.0)	1 (0.9)	0 (0.0)

FABQ: Fear-Avoidance Beliefs Questionnaire; PCS: Pain Catastrophizing Scale; TSK: Tampa Scale of Kinesiophobia.

Floor or ceiling effects were considered present if more than 15% of participants achieved the minimum or maximum possible score.

## Discussion

This prospective measurement-property study evaluated and compared the responsiveness of the Turkish versions of the FABQ, TSK and PCS as the primary psychological outcome measures in adults with chronic NSLBP. It also derived GRC-defined MIC estimates. The measures showed distinct longitudinal profiles. FABQ change corresponded strongly with GRC-defined improvement, PCS showed more modest correspondence and greater uncertainty around its MIC estimates, and TSK changed substantially by distribution-based indices without distinguishing participants who reported global improvement. These findings show that the magnitude of observed change and its correspondence with patient-perceived recovery are related but distinct aspects of longitudinal interpretation.

The strong FABQ-GRC correspondence is consistent with previous rehabilitation studies [15,17]. Beliefs about physical activity and work may contribute directly to patients’ judgments of function and recovery. However, the rehabilitation program included education addressing fear, avoidance, safe movement and the perceived threat of pain, and was therefore closely aligned with constructs represented by the FABQ. The findings indicate stronger FABQ-GRC correspondence in this rehabilitation context rather than inherent superiority of the FABQ across interventions. The GRC-defined MIC estimates were 5 points for FABQ-Physical Activity and 7 points for FABQ-Work, with relatively narrow bootstrap intervals. Findings from the employed-only analysis were consistent with those from the full cohort, suggesting that inclusion of non-working participants did not explain the strong FABQ-Work result. Nevertheless, these estimates remain sample- and setting-specific.

PCS change showed moderate correspondence with GRC-defined improvement and lower discrimination than FABQ change. Its bootstrap intervals were also wider, particularly for PCS-Total [MIC estimate, 6 (95% bootstrap CI 3–11)]. This pattern partly accords with previous evidence that the PCS is responsive in chronic LBP rehabilitation [16], while indicating greater uncertainty in the present cohort. Catastrophizing includes cognitive and emotional responses that may change without being fully reflected in a global judgment of recovery. The PCS MIC estimates should therefore be interpreted as preliminary, context-specific estimates and confirmed in independent samples.

TSK scores showed substantial change by distribution-based indices, but change was unrelated to the GRC and discrimination was at chance level. This contrasts with findings for Italian [34] and Persian versions of the TSK [17] and may reflect differences in intervention content, anchor definition, baseline severity, follow-up period or cultural context. The result does not imply that kinesiophobia is clinically unimportant. Rather, it indicates that change in this construct did not correspond to the broad global recovery judgment captured by the GRC in this cohort. The TSK may remain useful for monitoring fear of movement, but its change score should not be used alone to infer global improvement, and the negligible anchor association did not support estimation of a GRC-defined MIC.

Cultural and occupational context is relevant when interpreting all three measures, particularly FABQ-Work. Beliefs about pain, movement, work and recovery may be shaped by family and social expectations, employment conditions, and healthcare experiences. In Turkish-speaking populations, the meaning of work may also extend beyond paid employment to domestic responsibilities, caregiving or anticipated return to work. Linguistic validation alone does not ensure that responsiveness findings or MIC estimates transfer across settings. These estimates should therefore be applied with attention to the target construct, occupational context, intervention and cultural setting, and should not be treated as universal thresholds.

The study’s strengths include its prospective design, direct comparison of three commonly used measures within the same cohort, and complementary use of distribution-based indices and an external patient-reported anchor. Bootstrap CIs quantified uncertainty around the ROC-derived MIC estimates, and the employed-only analysis addressed an important interpretive concern regarding FABQ-Work. The findings extend evidence for the Turkish versions of these measures from cross-sectional validity and reliability to longitudinal responsiveness and interpretation.

Several limitations should be considered. The GRC was a retrospective global anchor rather than a construct-specific assessment and may have been influenced by current symptoms, recall, expectations or satisfaction with care [30,35]. Accordingly, the MIC estimates represent correspondence with GRC-defined improvement rather than universal thresholds for change in fear-avoidance beliefs, kinesiophobia or catastrophizing. Participants with GRC scores of 3–5 were classified as not improved, not stable; because only 16 participants selected GRC = 4, a robust stable-sample assessment of reliability and measurement error was not possible. In addition, non-working participants received no standardized frame of reference for FABQ-Work, although the employed-only findings were consistent with the primary analysis.

The rehabilitation program was used to induce change and was not designed to test treatment effectiveness; its alignment with fear-avoidance content may limit generalization to other interventions. Finally, recruitment from a single Turkish hospital limits generalizability to other settings and populations. Future studies should evaluate these measures using construct-specific anchors, adequately sized stable samples when measurement error is of interest, and independent cohorts representing different clinical, occupational and cultural contexts.

## Conclusions

In this rehabilitation cohort, FABQ change scores showed strong correspondence with GRC-defined improvement and yielded relatively precise MIC estimates. PCS change showed more modest correspondence and greater uncertainty around its MIC estimates, whereas TSK scores changed substantially by distribution-based indices but did not distinguish participants who reported global improvement; therefore, a GRC-defined MIC was not estimated for the TSK. These findings support selecting and interpreting pain-related psychological measures according to the target construct, intervention, anchor, occupational relevance and cultural context. The reported MIC estimates are sample- and context-specific and require external validation before broader clinical application.

## Data Availability

The data that support the findings of this study are available from the corresponding author upon reasonable request. The data are not publicly available due to privacy and ethical restrictions concerning participant-level clinical data.

## Open Scholarship

This article has earned the Center for Open Science badges for Preregistered. The materials are openly accessible at https://sreereg.icpsr.umich.edu/sreereg/subEntry/6020/pdf?section=all&action=download.
